# Addressing bias in big data and AI for health care: A call for open science

**DOI:** 10.1016/j.patter.2021.100347

**Published:** 2021-10-08

**Authors:** Natalia Norori, Qiyang Hu, Florence Marcelle Aellen, Francesca Dalia Faraci, Athina Tzovara

**Affiliations:** 1 Institute of Computer Science, University of Bern, Neubrückstrasse 10 3012 Bern, Switzerland; 2 Population Health Sciences, Bristol Medical School, University of Bristol, Bristol BS8 1UD, UK; 3 Institute of Digital Technologies for Personalized Healthcare (MeDiTech), Department of Innovative Technologies, University of Applied Sciences and Arts of Southern Switzerland, 6962 Lugano, Switzerland; 4 Sleep Wake Epilepsy Center | NeuroTec, Department of Neurology, Inselspital, Bern University Hospital, University of Bern, 3010 Bern, Switzerland; 5 Helen Wills Neuroscience Institute, University of California Berkeley, Berkeley, CA 94720, USA

**Keywords:** artificial intelligence, deep learning, health care, bias, open science, participatory science, data standards

## Abstract

Artificial intelligence (AI) has an astonishing potential in assisting clinical decision making and revolutionizing the field of health care. A major open challenge that AI will need to address before its integration in the clinical routine is that of algorithmic bias. Most AI algorithms need big datasets to learn from, but several groups of the human population have a long history of being absent or misrepresented in existing biomedical datasets. If the training data is misrepresentative of the population variability, AI is prone to reinforcing bias, which can lead to fatal outcomes, misdiagnoses, and lack of generalization. Here, we describe the challenges in rendering AI algorithms fairer, and we propose concrete steps for addressing bias using tools from the field of open science.

## The bigger picture

Bias in the medical field can be dissected along three directions: data-driven, algorithmic, and human. Bias in AI algorithms for health care can have catastrophic consequences by propagating deeply rooted societal biases. This can result in misdiagnosing certain patient groups, like gender and ethnic minorities, that have a history of being underrepresented in existing datasets, further amplifying inequalities.

Open science practices can assist in moving toward fairness in AI for health care. These include (1) participant-centered development of AI algorithms and participatory science; (2) responsible data sharing and inclusive data standards to support interoperability; and (3) code sharing, including sharing of AI algorithms that can synthesize underrepresented data to address bias. Future research needs to focus on developing standards for AI in health care that enable transparency and data sharing, while at the same time preserving patients’ privacy.

## Teaser

Artificial intelligence (AI) has an astonishing potential in revolutionizing health care. A major challenge is that of algorithmic bias. Most AI algorithms need big datasets to learn from, but several groups of the human population are absent or misrepresented in existing datasets. AI is thus prone to reinforcing bias, which can lead to fatal outcomes and misdiagnoses. Here, we describe challenges in rendering AI algorithms fairer, and we propose concrete steps for addressing bias using open science tools.

## Introduction

Despite the astonishing potential of artificial intelligence (AI) in health care, its regular use in the clinical routine comes with several ethical and societal challenges. As a notable example, one of the most frequent medical therapies is oxygen administration, whose levels in the blood are measured through a pulse oximeter.^1^ The pulse oximeter measures oxygen saturation by sending infrared light through the skin. Measurements of the pulse oximeter are known to be affected by the patient’s skin color, as the device systematically overestimates oxygen saturation levels in nonwhite patients.^2^ As a result, Black patients are three times more likely to suffer from an occult hypoxemia that remains undetected by pulse oximeters compared with white patients.^1^ As highlighted by this example, disparities in health care may start at the level of clinical measurements, which can ultimately shape erroneous medical decisions for entire patient groups, and can be amplified with the development of AI technologies.

AI promises to provide data-driven approaches to support clinical decision making and public health policymaking, gradually benefiting the health of society. Deep neural networks have generated substantial advances in medical imaging and precision medicine. In contrast to more “traditional” machine learning approaches, deep neural networks rely on propagating an input signal through multiple layers of transformations.^3^ This results in the extraction of more complex patterns of information from the input signal than simpler techniques are typically able to reveal. As the amount of data in the biomedical field constantly increases, the use of deep learning has also seen a vast increase, as deep neural networks are particularly powerful in extracting information from large datasets.^4^

In one of many examples, in the field of dermatology, convolutional neural networks (CNNs) are able to classify images of skin lesions as accurately as trained dermatologists.^5^ Notably, CNNs have even been found to be superior to dermatologists in melanoma image classification.^6^ In cardiology, machine learning has been proposed for developing risk assessments and performing predictions of cardiovascular events.^7^ In sleep medicine, deep learning can automate sleep scoring, a tedious task that is otherwise manually performed.^8^ Similar applications also have been reported in the fields of neurology, radiology, and pathology.^9^^,^^10^ Apart from an important role in diagnostics, AI also has applications in drug discovery and development, where it could be used to identify drug-drug interactions and to develop personalized treatments.^11^ AI systems could also help reduce health care costs, predict patients’ no show, or shorten hospital waiting times by searching millions of medical records.^12^ Our goal with this article is to focus on the question of AI and fairness in relation to bias in health care, and examine how open science tools can help address it. We start with an overview of known sources and examples of bias in the medical field. We then focus on data bias, and outline the main open challenges that need to be addressed from an algorithmic, medical, and societal point of view. Last, we offer recommendations for future directions, highlighting the role of open science in addressing bias in AI.

## AI and bias in medicine

**Figure 1:**
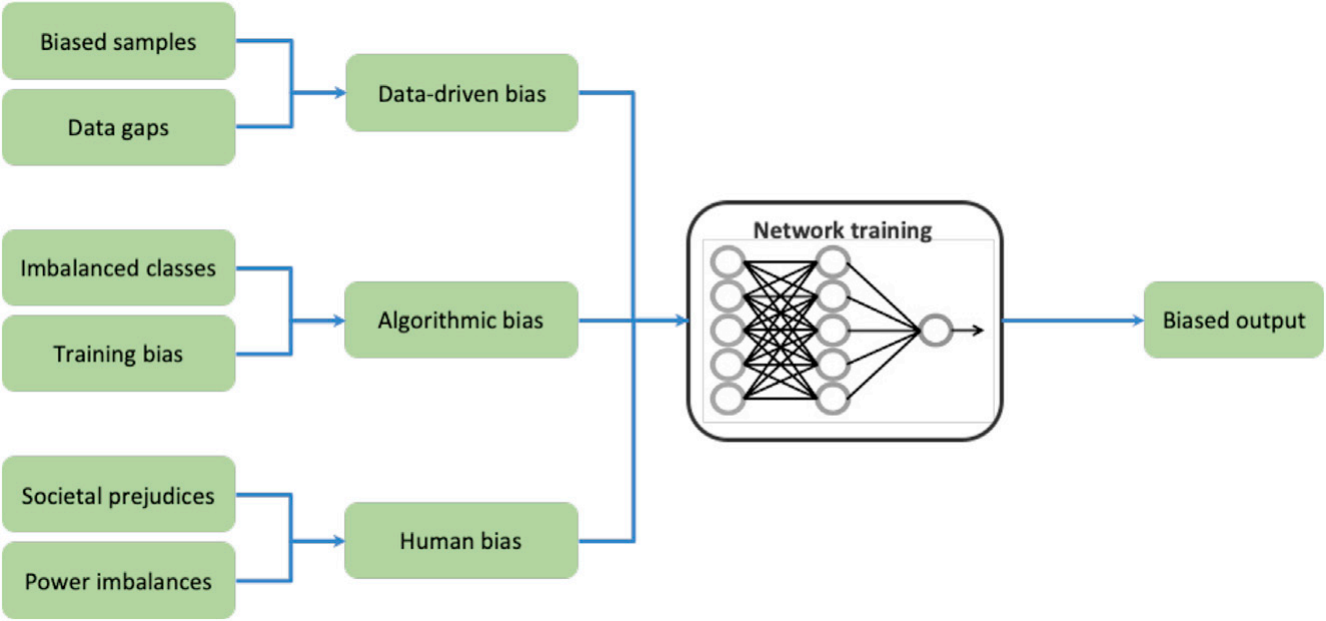
Illustration of different sources of bias in training machine learning algorithms

Bias can be defined statistically and socially. Statistically, bias refers to cases in which the distribution of a given dataset is not reflecting the true distribution of the population. Statistical bias can cause an algorithm to produce an output that differs from the true estimate.^13^ Social bias, by contrast, refers to inequities that may result in suboptimal outcomes for given groups of the human population.^12^ The medical field is no stranger to bias, which oftentimes is hard to quantify and detect (see Figure 1 for an overview). To date, there have been numerous reports of algorithms that discriminate against vulnerable groups in the same fields in which AI has shown promising results.

In one of many examples, CNNs that provide high accuracy in skin lesion classification^6^ are often trained with images of skin lesion samples of white patients, using datasets in which the estimated proportion of Black patients is approximately 5% to 10%.^14^ As a result, when tested with images of Black patients, the networks have approximately half the diagnostic accuracy compared with what their creators originally claimed.^14^ Black patients, whose lesions may have different characteristics from white patients, may thus be less likely to be accurately diagnosed by automated algorithms. This omission should not be taken lightly, as Black patients have the highest mortality rate for melanoma, with an estimated 5-year survival rate of only 70%, versus 94% for white patients. Misdiagnoses and socioeconomic barriers hindering access to health care may cause skin cancer at a more advanced stage in Black patients, hindering treatment.^15^

Racial bias in health care results in some groups of patients getting better medical treatment than others. In another example, AI algorithms used health costs as a proxy for health needs and falsely concluded that Black patients are healthier than equally sick white patients, as less money was spent on them.^16^ As a result, these algorithms gave higher priority to white patients when treating life-threatening conditions, such as diabetes and kidney disease, even though Black patients have higher severity indexes.^16^

The Coronavirus Disease 2019 (COVID-19) has proven how biased AI systems amplify existing inequalities, placing vulnerable populations at a higher risk of severe illness and death.^17^ AI is used in the fight against COVID-19, but because of the time pressure to develop concrete solutions against the pandemic, AI might be likely to reinforce COVID-19–induced inequalities at scale, because its performance in vulnerable populations may not have been thoroughly tested.^18^

Algorithmic bias is not exclusive to race. Gender inequalities also can be exacerbated by imbalanced algorithms. For example, in cardiology, a heart attack is overwhelmingly misdiagnosed in women.^19^ Nevertheless, prediction models for cardiovascular disease that claim to predict heart attacks 5 years before they happen^20^ are trained in predominantly male datasets. As cardiovascular disease has different patterns of expression in men versus women,^21^ an algorithm that has been trained predominantly with data samples of men may not be as accurate in diagnosing women.

Another interesting area for AI in medicine is the quest for automated sleep scoring algorithms. Since the 1960s, many algorithms have been developed, reaching very good perfomances,^22^ but when used in a clinical routine they fail miserably. Being trained on young healthy individuals, automated algorithms are often unable to decrypt sleep disorders in older patients. Thanks to bigger and more heterogeneous datasets, automated sleep staging has improved, but we are still far from acceptable performance when validation is done on new datasets or unseen sleep disorders. In sleep scoring, training of AI algorithms is done utilizing visual scoring labels as gold standards. Cognitive biases can lead to poor inter- (70%–80%) and intra- (90%) scoring agreement.^23^ This intrinsic limit could be overcome by AI, as its use neutralizes external sources of variance like human expert variability, offering a uniform standardized solution.^8^

Bias is concerning in areas where the lack of variability in training data is harder to identify at an early stage, such as drug development and clinical trials. In the case of clinical trials, the majority of participants are male, of a limited age group, and from similar ethnic backgrounds.^24^ Preclinical studies are also affected by gender bias, as they typically include either a vast majority, or exclusively male animals,^25^ which resulted in the NIH issuing guidelines to balance the ratio of male/female animals.^26^ Gender biases during the preclinical stages of drug development could alter how women react to newly developed drugs.^27^ The results of drug behavior, side effects, and effectiveness from such early studies may in turn be transferred into the datasets that are then used to train AI algorithms.

Data limitations are a critical issue that can result in bias (Figure 1), but the lack of diversity in clinical datasets is not the only source of bias. Researchers and clinicians can also impute unconscious judgments and biases into their research (Figure 1), thus deploying AI algorithms that are biased by design. If ethical issues are not addressed before further implementation of algorithms in the clinical practice, AI might fail to deliver benefits to all patients, increasing health inequities.

## Moving toward fairness in AI: Current challenges

### Bias

Sources of bias in AI may be present in most, if not all, stages of the algorithmic development process. Algorithmic bias can emerge due to the use of imbalanced or misrepresentative training data, the implementation of data collection systems influenced by human subjectivity, lack of proper regulation in the design process, and replication of human prejudices that causes algorithms to mirror historical inequalities.^13^

Vulnerable groups have a long history of being absent or misrepresented in existing datasets. When AI algorithms are trained with datasets in which vulnerable groups are not well represented, their predictive value may be limited. Algorithms may be able to detect patterns specific to the majority groups that they were trained with, but they may have poor performance in recognizing patterns that are present in patient groups that were never seen during training. As an example, skin cancer has a strong genetic component.^15^ If a diagnostic algorithm is only trained with genetic data of white patients, it may fail to generalize to patients of other ethnicities.

More generally, if AI is used as a diagnostic or therapeutic approach in patients who are invisible in the datasets that AI algorithms are trained with, these may fail to diagnose or treat entire patient groups, such as ethnic and gender minorities, immigrants, children, the elderly, and people with disabilities. These failures for certain population groups can be hard to recognize during the early training and testing phases of AI deployment, unless they are specifically sought after.

#### Sources of bias

##### Data-driven bias

Most fields of human research are heavily biased toward participants with a Western, Educated, Industrialized, Rich, Democratic—WEIRD—profile,^28^ and are not representative of the human population as a whole. As several of the available datasets that are used to train AI algorithms were collected in the context of scientific studies, they in turn are biased.

Oftentimes, the quantification of certain forms of bias in a given dataset is relatively straightforward, as the data samples carry features that reflect the characteristics of bias. For example, bias due to ethnicity could be inferred from a dataset of skin samples, or bias due to gender or ancestry can be inferred from genetic data. However, in many cases it is impossible to quantify biases in the composition of a dataset. For instance, biases due to socioeconomic status or sexual orientation are often impossible to infer in a biomedical dataset unless this information has been explicitly collected and included as metadata.

Although variables and metadata that do not directly apply to a given research question may seem irrelevant for quantifying bias, there is strong evidence that suggests the contrary. For instance, several neuroscience studies have shown that socioeconomic variables are associated with detectable differences in brain structure^29^ and functions.^30^ To be able to assess the influence of socioeconomic variables in neurological data, future studies will need to start collecting homogenized metadata corresponding to factors that may induce bias.

As an example of data-driven bias and data gaps, polygenic risk scores use data from genome-wide association studies (GWAS) to calculate a person’s inherited susceptibility for a disease. Although polygenic risk scores have a great potential as predictive biomarkers, 81% of GWAS studies are conducted in individuals of European ancestry.^31^ This affects the generalizability of polygenic risk scores across different populations and can result in biased predictions and further inequities in health outcomes.

##### Algorithmic bias

When an algorithm is trained on biased data, it is likely to reinforce patterns from the dominant category of the data it was trained with. In the simplest case, when an algorithm is trained to classify a dataset consisting of 80% healthy and 20% diseased images, just by predicting every sample as healthy, the algorithm will achieve a performance of 80% accuracy. Alternative metrics should therefore be used that are attuned to class imbalance, such as the F1 score. This can be defined as follows:

$$F1\;=\;\frac{TP}{TP+\frac{FP+FN}{2}}$$

where TP = true positive, FP = false positive, and FN = false negative. In the above-mentioned example of an imbalance dataset, this would result in a score of 0. Therefore, the F1 score could be a more reliable and intuitive metric in the case of imbalance datasets.

Having objective ways to estimate chance levels is crucial to avoid misinterpretation of findings. Permuting the labels of the available samples and retraining an algorithm to give “random” predictions can provide an empirical estimation of chance levels.^32^ This should be combined with performance metrics that are not affected by imbalanced datasets,^33^ or with classification techniques that include weight factors that take into account in the algorithms’ optimization step the fact that some classes are imbalanced.^33^

Moreover, algorithms that mitigate bias can be used whenever possible. To increase algorithmic fairness, protected attributes, such as gender or ethnicity, can be included during training in order to ensure that algorithmic predictions are statistically independent from these attributes.^34^ Alternatively, loss functions can be defined per protected group, and may be forced to remain below a certain level for all defined groups, so that no single group is systematically misclassified.^34^ Similar approaches have been introduced in different AI frameworks, such as in the case of adversarial learning,^35^ and are summarized in open-source toolkits that can be used to mitigate algorithmic bias.^36^

##### Human bias

As AI algorithms are designed by humans, they may often reflect human biases. Algorithms are often designed to tackle what their developers consider the most urgent problems to solve, which are not necessarily the same challenges faced by the individuals that are concerned by those algorithms. Lack of diversity in engineering and biomedical teams can replicate unconscious bias and power imbalances.^37^

Human bias in AI can be one of the hardest ones to detect and mitigate, as it can result from long-held societal prejudices that may be subtle at the level of society, and amplified by AI and large datasets. The medical field has several examples where racial, gender, or age disparities are affecting clinical decision making, quality of treatment, and outcome prognosis.

It is well documented that Black patients have lower survival rates compared to white patients for different cancer types.^38^ Although mortality rates from cardiovascular disease have majorly decreased over the past 10 years, Black patients had higher mortality rates in 2017 compared with the mortality rate that white patients had back in 2007.^39^ Similar trends are seen for patients suffering from depression, with ethnic minorities experiencing more severe symptoms and receiving medication less often than white patients.^40^

Apart from race, also gender results in bias and unequal treatments. Historically, women have been regarded as the “smaller” version of men, and medication dosages were adjusted for patient size, without taking into account sex differences.^41^ In health care, sex differences can be substantial and include differences in gene expression,^42^ or in the prevalence, age, onset, symptomatology, morbidity, and mortality of life-threatening diseases, such as coronary heart disease,^43^ stroke, and different types of cancer.^44^ Compared with men, women are more likely to have their pain levels underestimated by clinicians.^45^ Moreover, nonheterosexual women exhibit higher risk factors for certain forms of cancer, cardiovascular disease, or mental health, despite generally higher socioeconomic status than heterosexual women.^46^ Individuals who are lesbian, gay, bisexual, transgender, transsexual, two-spirit, queer, or questioning (LGBTQ+) are particularly affected by inequalities in health care, which arise due to particular needs for treatment^47^ and due to bias in health care practitioners.^48^

### Data gaps

Over the past decade, governments, funders, and institutions have worked together to promote open data sharing. As a result, the world has access to public datasets to train AI algorithms, but most of them are not diverse, disaggregated, and interoperable.^49^ Data repositories have substantially increased the number of open datasets available to train and develop algorithms, but vulnerable populations remain underrepresented in health care data. This lack of diversity restricts the utility and generalizability of the datasets and the AI algorithms trained with them. In addition, lack of consistency and coherency, differences in formatting, and limited data disaggregation prevent open datasets from being intermixed and used to power large, complex systems.

Developing inclusive technologies relies on counting people in, but gaps in data tend to leave certain groups unnoticed. When minority groups are invisible in datasets used to deploy AI algorithms, their needs and phenotypes may become invisible. As an example, commercial, and also open genomic databases, like the Personal Genome Project, contain data that are in their vast majority of European origin.^49^ The lack of genetic data for large parts of the human population might hinder the development of biomarkers and treatments for conditions with a heavy genetic component.

To characterize datasets, it is important to collect comprehensive metadata. As an example, despite numerous initiatives to include sexual orientation and gender identity in electronic health records, to date this information is largely missing.^50^ In the vast majority of medical records it is thus impossible to identify LGBTQ+ individuals, who experience health disparities, and may have unique health care needs. Moreover, information related to the researchers or clinicians who collected certain metadata is also often missing, and its importance is ignored. Labels associated with medical data, disease rating scales, and diagnosis may be imbued with cognitive biases of the health care personnel who collected this information.^51^ Personality traits like tolerance to risk, or overconfidence may result in diagnostic, therapeutic, or management errors, and may impact patient outcomes.^44^

### Data standards and interoperability

Standardization makes data interoperable and impactful. When data are not openly available and are published in inconsistent and incompatible formats, it becomes difficult to exchange, analyze, and interpret them. Inconsistency in data sharing, variability in data quality, and different levels of data usability determine whether or not researchers get access to high quality training datasets for fair AI.^52^

Importantly, several of the underlying datasets that power AI algorithms were not built for this purpose. As a result, the data standards (or lack of) applied to these datasets limit the potential of the algorithms that are trained with them. This is a major limitation in many of the datasets published in data science websites, such as Kaggle, where binary gender fields, incomplete gender disaggregation, and incompatible formats make it difficult to not only build inclusive AI, but also to test for biases.

Apart from formatting, data standardization can encourage patient groups to capture their characteristics in a way that facilitates readability and interoperability. For data standards to reflect those who are ultimately impacted by their adoption, broad and active participation from members of different sectors and communities is required during all steps of their design process.

To become interoperable, datasets need to track and measure inclusivity, have the possibility to exchange samples, and have clear structures that are capable to support multiple systems. Creating data standards is a complex process, but also a mandatory point of passage for training fair AI algorithms. In the fast-paced field of AI, it sometimes might be better to adopt existing standards instead of creating completely new ones.

## Moving toward fairness in AI: A call for open science

The fair implementation of AI in health care requires integrating principles of inclusivity, openness, and trust in biomedical datasets by design. The idea of openly sharing multiple facets of the research process, including data, methods, and results under terms that allow reuse, redistribution, and reproduction of all findings gave birth to open science, a practice that is strongly supported by several institutions and funding agencies.

**Figure 2:**
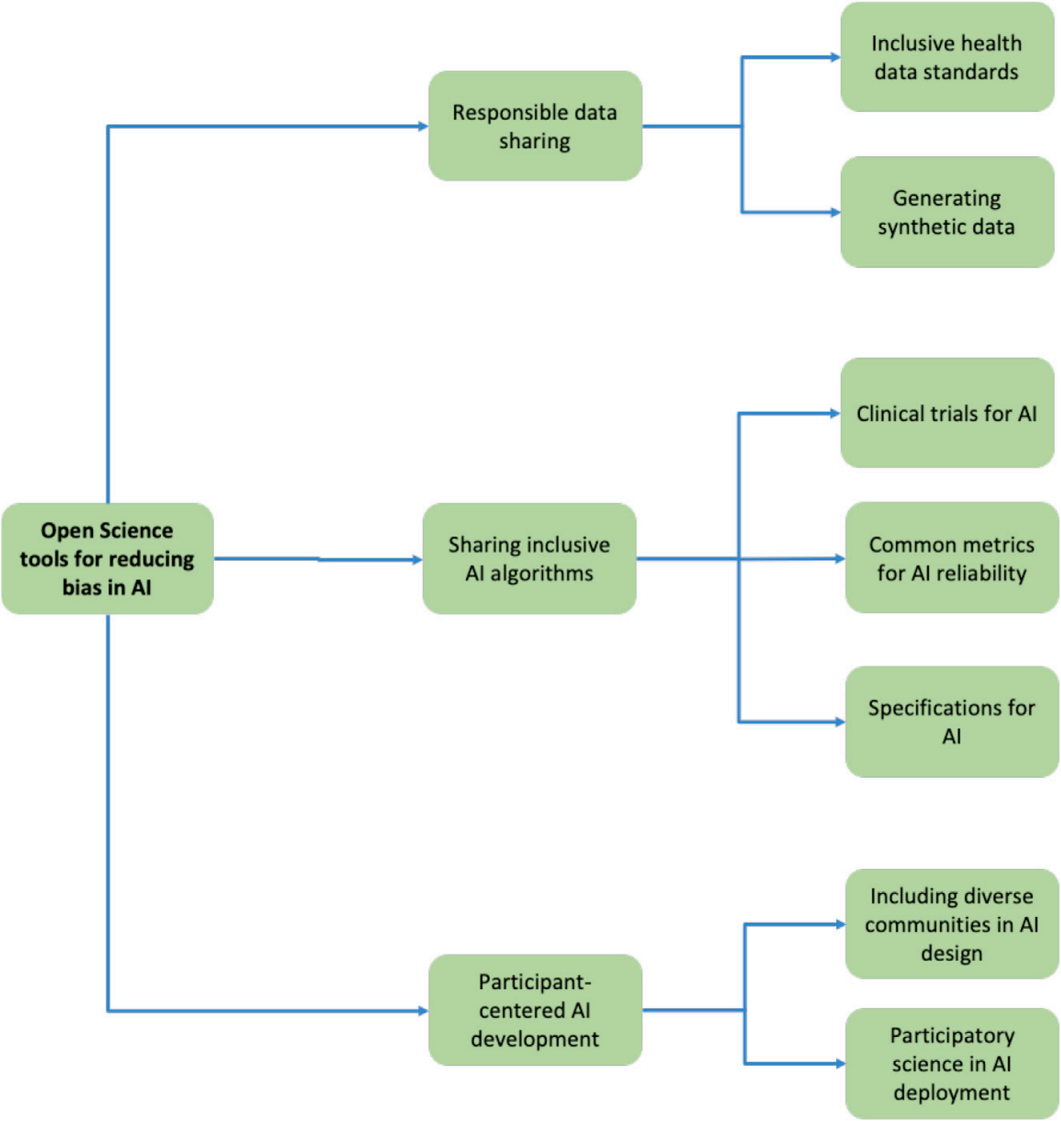
Illustration of open science tools that can help address bias in AI

Although open science is a wide term that encompasses several practices, recent attempts have framed open science within a framework of inclusivity, such that no science can be open unless it is inclusive by design. This inclusivity aspect, along with the well-established advantages of increasing scientific rigor, trust, and use of resources, make open science suitable for increasing algorithmic fairness in the biomedical field. Openly sharing the entire research process along with its results incentivizes participation and helps remove key barriers that prevent members of vulnerable or underrepresented groups from being part of the global scientific community. This can be achieved across several axes (see Figure 2 for a summary illustration).

### Sharing data increases inclusivity

During the recent years, the value of openly sharing health care data has become more evident than ever, with researchers, governments, and nongovernmental organizations worldwide implementing open data sharing practices to quantify and respond to health emergencies.^53^ The millions of data points being shared have accelerated the development of cutting-edge AI technology for epidemiologic, diagnostic, and therapeutic interventions. Although health care data are necessary to advance medicine, they contain sensitive information that needs to be safeguarded for privacy reasons.^54^ It is not enough to simply code the name and surname of a patient to ensure anonymity. In one of many examples, electroencephalography signals, which are typically considered anonymous, can be used as a biometric identifier.^55^ Therefore, new anonymization processes must be conceived. Responsible data sharing frameworks designed with openness at their core that also protect the individual’s rights to privacy are needed for health care data. One example of such a framework is the federated learning systems, which enable the training of AI algorithms at a local level, allowing individuals to maintain control and anonymity of their data.^56^

#### Inclusive health data standards to support interoperability

Data standards lead to efficient data infrastructure and support interoperability. Shared formatting, language, and data identifiers make information scalable, while comprehensive metadata descriptions enhance the discoverability of communities and concepts.^57^

Fair biomedical data standards cannot be developed in isolation, and require constant feedback from patient or community representatives.^58^ However, creating standards from the ground up is a complex process; therefore, the adoption of existing health data standards is advised. A number of existing health data standards are recommended by health authorities, such as Health Level 7, the International Organization for Standardization (ISO), and OpenEHR, among others.^58^ In addition, the Open Standards for Data Guidebook provides a general introduction to open data standards for data, making it easier to find and adopt existing standards and, when necessary, create new ones.

#### Generating synthetic data to combat bias

Oftentimes, despite the best intentions, it is impossible to have unbiased datasets. Questions of privacy, anonymity, and trust may obstruct the participation of underrepresented groups in data sharing initiatives. To overcome this limitation, the field of machine learning has several tools, such as Generative Adversarial Networks,^59^ that can be used to artificially generate synthetic data and augment underrepresented classes, such as skin lesion images.^60^ This can allow neural networks to be trained with more samples of data that may be underrepresented, such as data of ethnic minorities. Future studies can evaluate the efficacy of this approach in decreasing the rates of misclassified samples of underrepresented groups.

### Sharing inclusive AI algorithms

Sharing data is not always feasible or desirable due to questions of privacy and security. Thus, sharing code and retraining existing algorithms with data collected at a local level, for example in hospitals across the globe, can circumvent the lack of diversity in existing openly shared datasets.

Opening up the source code of AI algorithms can accelerate algorithmic development by allowing scientists and engineers to extend, reuse, and validate shared code. Open-source practices facilitate collaboration, making code and algorithms accessible to anyone, including members of sensitive groups.^61^ Openly sharing AI algorithms in a comprehensive way contributes to computational transparency and interoperability.

Sharing code can empower individuals to evaluate the performance of novel AI algorithms on different datasets. This can allow researchers from all around the globe to test whether a given algorithm, developed, for example, in Europe predominantly with data of white patients, generalizes to data of patients in Asia or Latin America. Sharing code can enable local research communities to validate and fine-tune existing neural networks for the needs of their local patient groups, resulting in a distributed model for training future AI algorithms.

#### Evaluating algorithmic efficiency and fairness

Field-testing can give researchers the opportunity to assess the performance of algorithms in different population groups and clinical settings.^62^ Given the ethical implications of AI in medicine, AI algorithms should be evaluated as rigorously as other health care interventions, like clinical trials.^63^ Open science practices that encourage transparency, like preregistration for AI studies, need to become the norm before these can be used to diagnose or treat a specific patient group. Moreover, transparent guidelines like the Good Evaluations and Practices for Health Informatics, can guide users through a multistep process to control for issues that may arise during different stages of algorithmic design and implementation.^64^

The limitations of AI algorithms that can be identified through such investigations should be transparently communicated to clinicians and policymakers. This can ensure that AI algorithms can be applied to the populations they have been tested on.

#### Common metrics for AI reliability

Another important issue is related to the inconsistency and limits of the metrics adopted for assessing AI reliability. The adoption of common standardized metrics should be strongly favored, and the clinical perspective should be considered in algorithmic applicability and interpretability. Whenever possible, the metrics should not only focus on the numerical accuracy, but also include quality of care and patient outcomes.^65^

#### Explainable AI models

A direction that AI algorithms will need to consider is that of explainable AI. Several powerful AI algorithms are employing a so-called “black box” approach, where it is difficult or even impossible to understand how the obtained results have been achieved.^66^ Explainable AI by contrast includes interpretable AI models, where the strengths and weaknesses of a decision-making process are transparent.^67^ AI applications often have to deal with a trade-off between model performance and interpretability. On the one hand, simple models, such as linear classifiers or decision trees, are generally interpretable but oftentimes lead to suboptimal performance. On the other hand, more complex models like deep neural networks provide high classification performance, but identifying the features that drive an accurate classification can be cumbersome and oftentimes impossible.

Feature interpretability, together with a strong performance are prioritized in explainable AI models. In explainable AI, the features that a model is using to make a decision need to be traceable and understandable by a human. As an example, transparent techniques like decision trees, relying on interpretable features, can provide a “white-box” approach for diagnosis.^68^

The field of computer vision has dedicated a substantial effort in obtaining interpretable features and understanding the process of classification.^69^ For example, the kernels or intermediate features of a trained neural network may shed light on the learned structure in different layers of the network, giving rise to methods like class activation mapping (CAM).^67^ Other methods are gradient based, like saliency maps,^68^ and calculate the contribution of each input pixel to the overall classification performance. The combination of these two approaches has given rise to Grad-CAM,^70^ which allows the identification of regions of interest in the input data that mostly influenced the network’s decision. These approaches can be integrated in the future with existing algorithms and datasets, so that features driving a network’s decision can be potentially shared together with the data used to train the network in order to increase transparency.

### Participant-centered development of AI algorithms

An important component of open science that can be a strong asset in the fight against bias in AI applications is participatory science. Participatory science involves scientists and nonscientists working together toward the creation of scientific knowledge.^71^ Participatory science can be used in the development of novel AI algorithms to actively include individuals who are concerned with the applications of a given algorithm, like specific patient groups. When members of underrepresented groups are actively engaged in science, they can contribute to the identification of bias against their communities, and with solutions to increase their representations in the datasets used to develop AI algorithms.^61^

Including communities (such as indigenous peoples, people with disabilities, the LGBQ + community, immigrants, etc.) in the design of data collection and AI deployment can ensure that the outcomes that can be achieved from the design of AI models directly benefit them. Moreover, the active engagement of patient groups in AI deployment might reduce the propagation of biases and misconceptions, and can help scientists evaluate whether their research questions are equally relevant to patients and groups that are traditionally underrepresented in science.

As a notable example, the Open Artificial Pancreas (OpenAPS) is a community-led initiative that designs openly accessible technology for automatically adjusting insulin intake in patients with type 1 diabetes, in order to keep blood glucose in a safe range.^72^ OpenAPS has resulted in patient-led data commons and in the generation of rich clinical datasets that may be used for patient-led research, and have already resulted in several research studies.^61^

Participant-centered algorithms and datasets can be facilitated by community-based platforms specifically designed to enable collection of personal data and give individuals the possibility to design novel study questions or algorithms that concern themselves and their communities.^61^ Open Humans is an example of such a platform that allows participants to share their personal data, design their own research questions, and also design and share their own algorithms. Open Humans takes a participant-centered approach to data sharing, in order to solve some of the challenges associated with data ethics, privacy, and patient involvement.^61^

## Conclusions

Health care is being transformed by the growing number of data sources that are constantly shared, collected, and implemented into AI systems. Using AI for public good can help tackle some of the world’s most pressing issues, including providing humanitarian assistance and supporting emergency response. One example of this is the United Kingdom’s National Health Service Covid-19 contact-tracing app, which helped prevent between 100,000 and 900,000 Covid-19 infections from October to December 2020.^73^ Organizations like Omdena and the Alan Turing Institute are pioneers in developing ethical AI solutions in a humanitarian context. From predicting climate risks, to increasing transparency, and responding to epidemics, these organizations have proven that when AI is inclusive and fair, it can be used in solving the world’s most pressing issues.

In order for new technologies to be inclusive, they need to be accurate and representative of the needs of diverse populations. Algorithmic and human bias, along with information gaps and lack of data standards, common metrics, and interoperable frameworks pose the biggest threats to move toward fair AI. Implementing the principles of open science into AI design and evaluation tools could help strengthen collaborations between the AI and medical fields, and open up the space for diverse voices to participate in AI deployment for medicine.
